# A Single Step Impression Technique of Flabby Ridges Using Monophase Polyvinylsiloxane Material: A Case Report

**DOI:** 10.1155/2014/104541

**Published:** 2014-04-27

**Authors:** Umesh Y. Pai, Vikram Simha Reddy, Rushad Nariman Hosi

**Affiliations:** ^1^Department of Prosthodontics, Manipal College of Dental Sciences, Manipal University, Manipal, Mangalore, Karnataka 575001, India; ^2^Department of Prosthodontics, A B Shetty Memorial Institute of Dental Sciences, Deralakatte, Mangalore-575018, India

## Abstract

Complete denture fabrication in clinically compromised situations such as flabby ridges is a challenging task for the clinician. Accurate impressioning of these tissues plays a major role in ensuring a well-fitting prosthesis. In this paper, the authors have proposed a newer technique of impression making of the flabby tissues using a combination of readily available newer and older materials to ensure an accurate and easy impression of these tissues.

## 1. Introduction

Construction of complete dentures and its performance in function depend on accurate impression of the denture bearing and limiting areas. The challenge to ensure adequate function increases manifold when confronted with denture bearing areas that are not conducive to adequate function. Flabby tissues present a challenging clinical scenario for the clinician to ensure a well-fitting prosthesis. Flabby ridge also called as fibrous ridge or displaceable ridge is mobile soft tissue present on the superficial aspect of the alveolar ridge. Flabby ridge is predominantly seen in the upper anterior region and is commonly associated with features of combination syndrome, as mentioned by Kelly [1]. Earlier studies show that prevalence of flabby ridges vary in either arches, with edentulous maxillae prevalence being 24% and edentate mandibles 5% [2, 3]. Another reason for flabby tissue is unplanned and uncontrolled dental extraction [4].

Typically these “flabby ridges” are composed of mucosal hyperplasia and loosely arranged fibrous connective as well as more dense collagenised connective tissues. In the soft tissue, varying amounts of metaplastic cartilage and/or bone have been reported [5]. The lesser resilient tissues can displace the dentures under masticatory load leading to loss of peripheral seal causing poor retention of the denture.

Management of a flabby ridge is mainly by three approaches:

1. surgical removal of fibrous tissue prior to conventional prosthodontics,
2. implant retained prosthesis
  - fixed,
  - removable,
3. conventional prosthodontics without surgical intervention [6, 7].

A poor ridge is better than no ridge, which could be a sequel to surgical excision of the flabby tissues [8]. Each technique has its advantages and shortcomings. The advantage of the surgical technique is that it provides a firm denture bearing area. Its limitations include chances of decrease in vestibular height requiring an additional surgery of vestibuloplasty. It is contraindicated in patients who are unwilling to undergo a surgical treatment.

Implant prosthesis take the support from the underlying bone hence minimal or no support is needed from the tissue area. In terms of patient economics and time taken for the completion of procedure, the implant supported prosthesis has its drawbacks. Other factors that must be considered include surgery, discomfort and inconvenience, general health of the patient, and risk of surgical complications or implant failure [4].

A particular problem is encountered in the conventional impression making if a flabby ridge is present within an otherwise “normal” denture bearing area. If the flabby tissue is compressed during conventional impression making it will later tend to recoil and dislodge the overlying denture [2]. Thus, over the years, several impression techniques have been suggested for the impression of a flabby tissue ridge which will support the flabby tissue but at the same time will not displace it.

Prosthodontic literature has documented various impression techniques for overcoming the problem of the flabby ridge. Magnusson et al. [5] described a technique where two impression materials are used in a custom tray using zinc oxide and eugenol over the normal tissues and impression plaster over the flabby area. Crawford et al. [6, 7] described a two-tray impression technique where two trays are fabricated and impression is recorded with two different materials and is then oriented intraorally. Osborne [8] described the “window” impression technique with a custom tray made with a window over the flabby tissues. A mucocompressive impression is first made of the normal tissues with zinc oxide and eugenol using a custom tray. Once set, a low viscosity mix of impression plaster is then painted onto the flabby tissues through the window. Once set, the entire impression is removed. Watt and McGregor, recently revisited by Watson [9], described a technique where impression compound is applied to a modified custom tray. The thermoplastic properties of this material are then manipulated to simultaneously compress the “normal tissues,” while avoiding displacement of the “flabby tissues” using the same material and impression tray. Over this manipulated impression compound, a wash impression with zinc oxide and eugenol is made. While this impression technique is clearly less complex than the previous three described, the problem with all four techniques is that they rely on materials such as impression plaster, impression compound, and zinc oxide and eugenol. Many newer materials, such as polyvinylsiloxanes, are currently available in the market with varying consistencies and dispensing methods to suit the dental practitioner. The purpose of this paper is to describe two impression techniques used by the authors using readily available impression materials for recording the edentulous flabby tissue along with the normal tissues around it.

## 2. Clinical Report

**Figure 1:**
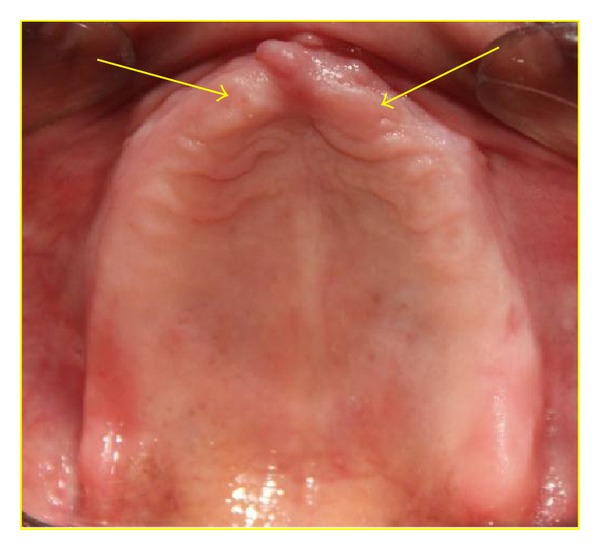
edentulous maxillary arch with arrows showing areas of flabby tissue.

**Figure 2:**
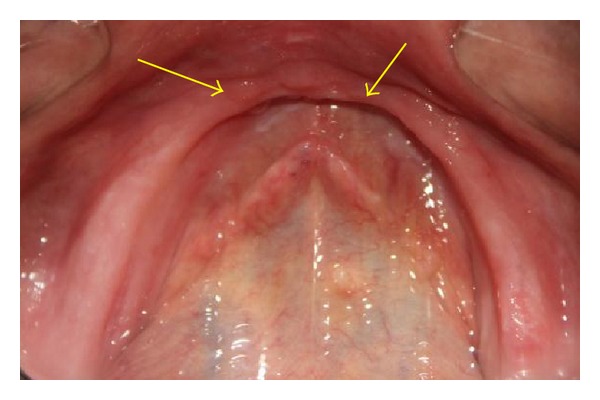
edentulous mandibular arch with arrows showing areas of flabby tissue.

A 68-year-old male patient came to the OPD of the Department of Prosthodontics with a chief complaint of replacement of missing teeth in upper and lower arches. The patient was a denture wearer for the last 5 years and described the existing dentures as “loose.” On examination the patient was completely edentulous in upper and lower arches. The anterior canine-canine region in maxilla along with the maxillary tuberosity and the mandibular canine-canine region was flabby (Figures 1 and 2).

The treatment options of implant supported prosthesis and surgical excision of the flabby tissue were suggested to the patient. The patient was not willing to undergo surgical procedures so it was decided that upper and lower complete dentures will be fabricated with a different impression technique.

A primary impression of the upper and lower arches was taken with alginate (Neocolloid; Zhermack) in the edentulous trays. The impressions were poured with dental stone and the displaceable tissues were identified on the cast.

**Figure 3:**
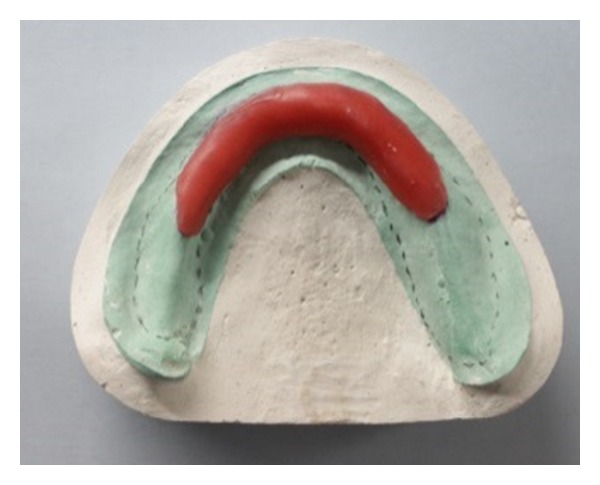
Additional wax placed to create relief over areas of flabby tissues.

**Figure 4:**
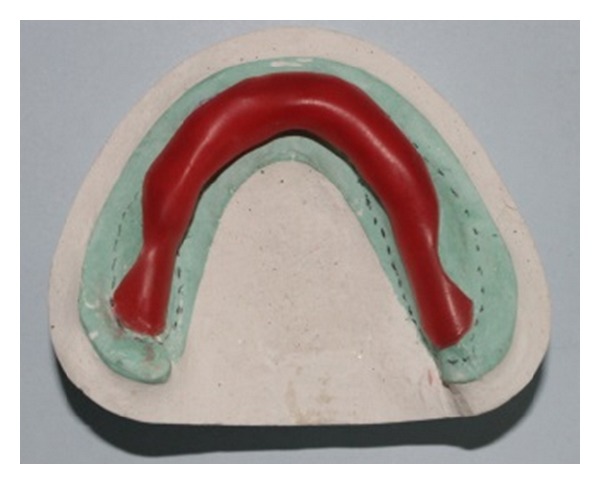
Relief wax placed over the mandibular arch.

**Figure 5:**
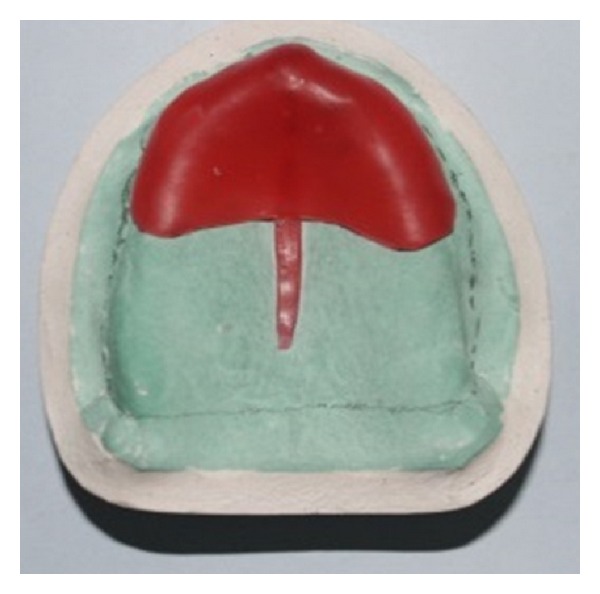
Relief wax placed over the maxillary arch.

On the maxillary cast, an “I” shaped spacer was applied along the mid palatine raphe using modelling wax with additional relief given in the flabby area from canine-canine region (Figure 5). The mandibular cast was first adapted with a layer of wax to provide extra relief in the flabby region (Figure 3) followed by addition of one more layer of wax covering the ridge except the buccal shelf area (Figure 4).

**Figure 6:**
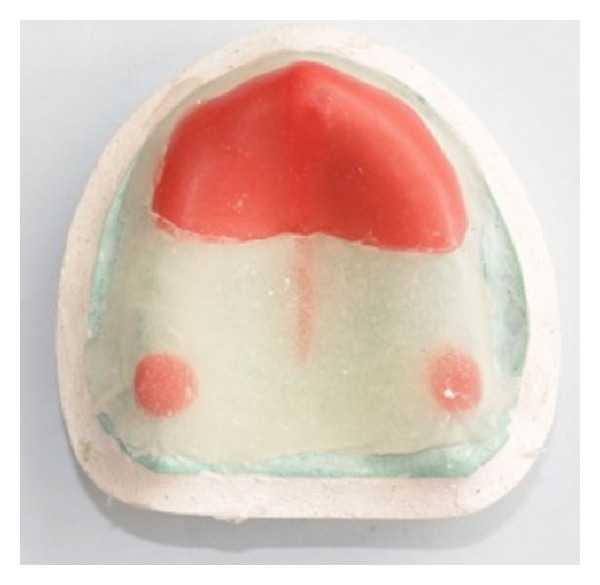
Clear acrylic custom “open” tray.

**Figure 7:**
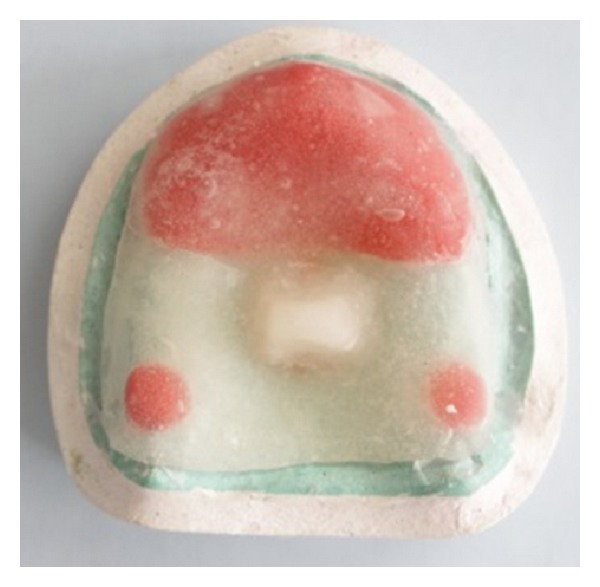
Maxillary custom tray with “supporting” tray covering areas of flabby tissue with the handle placed at the center of the palatal area.

**Figure 8:**
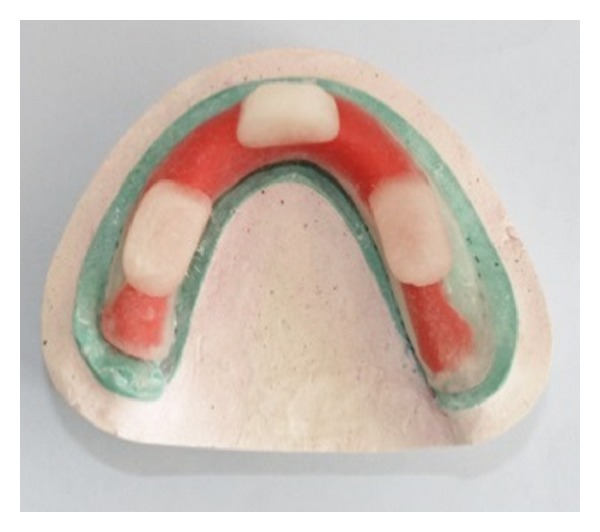
Mandibular custom tray fabricated with clear acrylic resin.

A maxillary custom tray was fabricated using clear autopolymerising acrylic resin (RR self-cure acrylic resin, Dentsply, India) covering the tissues except the area that was flabby (Figure 6). Over the “open” area of the tray another “supporting tray” of clear acrylic was made thus covering the flabby ridge. A mandibular custom tray was fabricated with autopolymerising clear acrylic (Figure 8). Clear acrylic resin was preferred for tray fabrication as tissue blanching underneath the tray could be easily evaluated, thereby making it easier for the operator to relieve pressure spots on the tray. The handle was placed in the palatal portion of the maxillary tray to ensure visualization of the underlying tissues through the clear acrylic tray and also to facilitate uniform distribution of pressure during impression making (Figure 7).

### 2.1. Final Impression of the Lower Ridge

**Figure 9:**
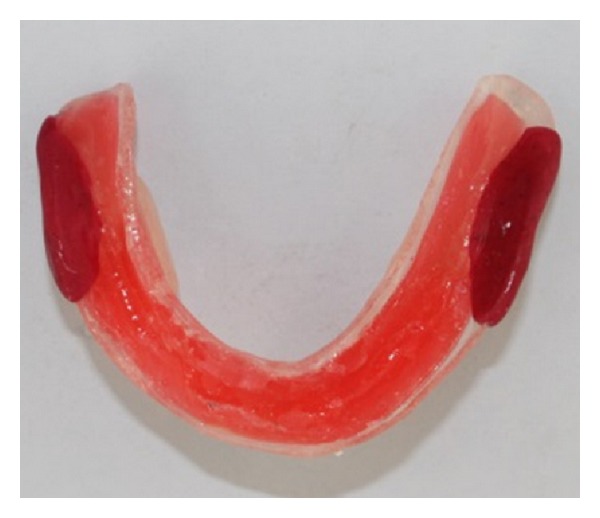
Buccal shelf area recorded with impression compound.

**Figure 10:**
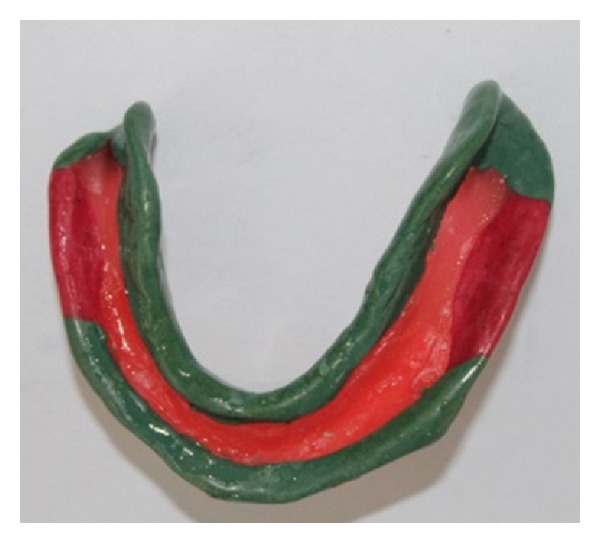
Custom tray with complete mandibular border moulding.

The Buccal shelf area was recorded by using mucocompressive impression material like impression compound Type 1 (Y Dents, Mumbai, India) (Figure 9). Lynch and Allen [10] advocated the use of impression compound over the buccal shelf area for recording impressions in distal extension partial denture ensuring a stable and uniform contact on the buccal shelf area, which in this case is the primary stress bearing area. It also acts as a stopper for the tray in the final impression procedure. The remaining borders of impression were recorded by selective pressure technique using green stick compound (Pinnacle tracing stick, DPI, Mumbai, India) (Figure 10).

**Figure 11:**
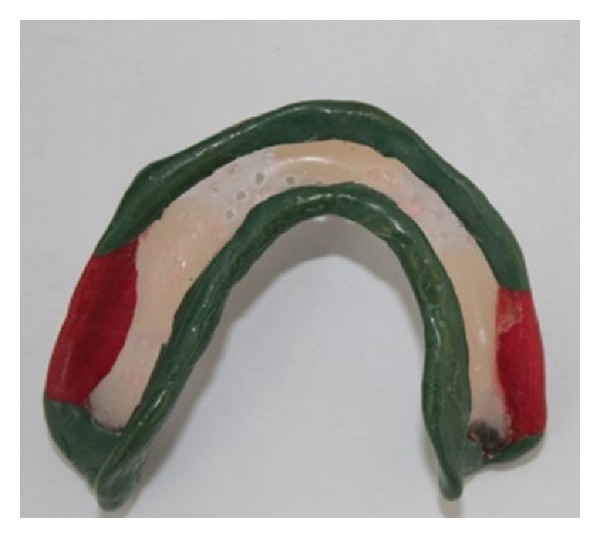
Mandibular custom tray with wax spacer removed. Multiple relief holes are placed in areas of flabby tissue.

**Figure 12:**
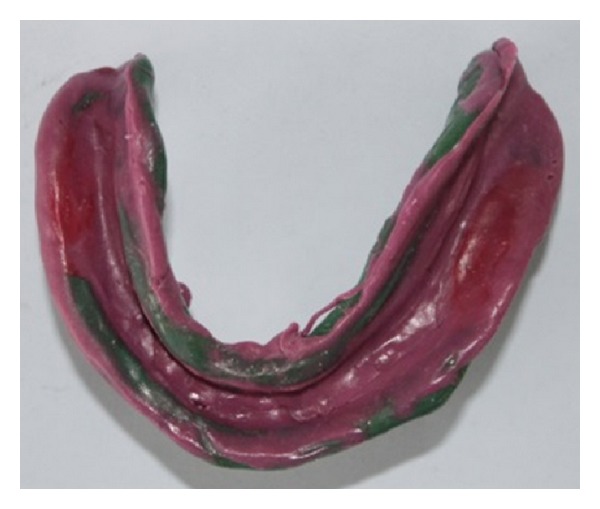
Completed mandibular impression with monophase polyvinylsiloxane material.

The spacer wax was then removed and multiple holes were drilled in the region of the flabby tissue. Tray adhesive was applied. A final impression with monophase (medium body) (Aquasil LV Monophase, Dentsply Caulk) addition silicone was made (Figure 11). A monophase impression material was preferred as it has the desirable thixotropic property thereby ensuring adequate flow under pressure (Figure 12).

### 2.2. Final Impression of the Upper Ridge

**Figure 13:**
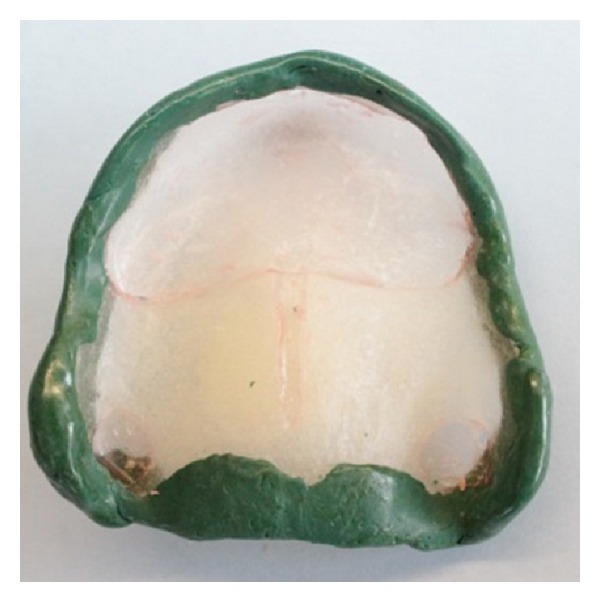
Maxillary custom tray with wax spacer removed.

**Figure 14:**
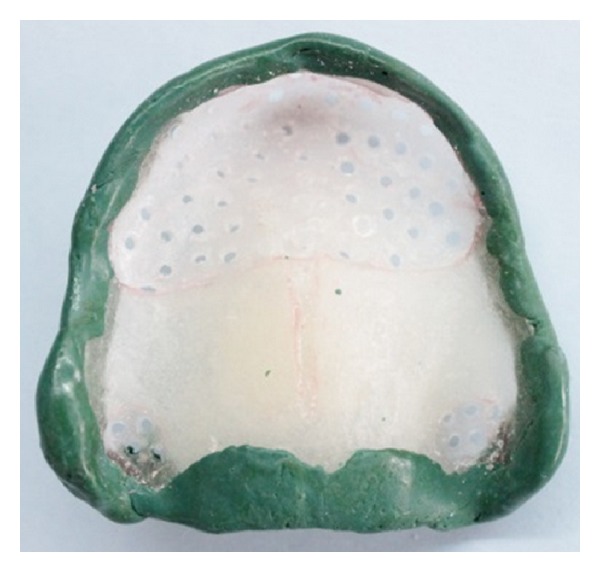
Maxillary custom tray with multiple relief holes.

**Figure 15:**
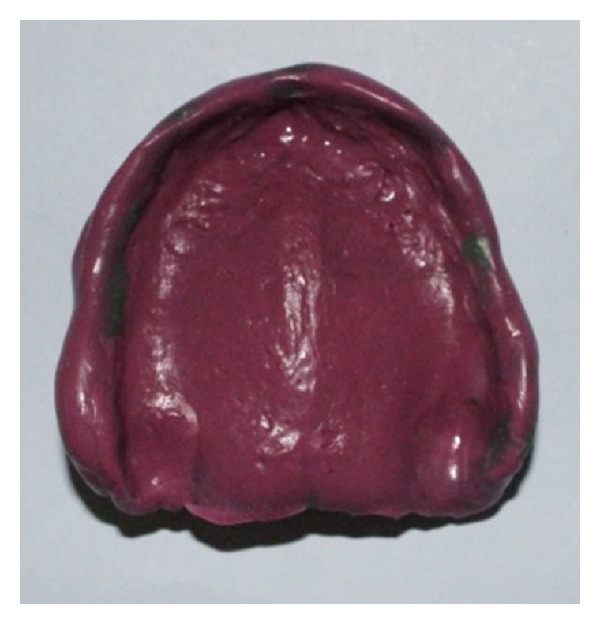
Completed secondary impression with monophase polyvinylsiloxane material.

The maxillary borders were recorded by selective pressure impression technique using green stick compound (Figure 13). The relief wax was removed and multiple holes were drilled in the “supporting tray (Figure 14).” Placement of multiple relief holes was done to ensure prevention of pressure build-up in the flabby area thereby leading to inadvertent tissue compression. Tray adhesive was applied. Similar to the lower impression a monophase impression of addition silicone was made (Figure 15).

**Figure 16:**
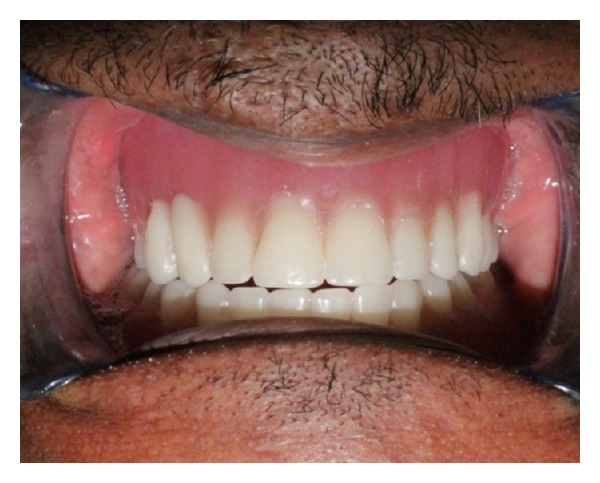
Fabricated prosthesis.

Subsequently, conventional treatment procedures were followed to deliver complete denture prosthesis (Figure 16).

## 3. Discussion

Due to the obvious difficulties in analysis of the success of prostheses constructed using the various impression techniques described, the clinical choice has fallen mainly to personal preference, based on analysis of theoretical principles. Various techniques have been recommended and there is controversy as to whether a mucodisplacive technique which compresses the mobile tissue aiming to achieve maximum support from it or whether a mucostatic technique with the aim of achieving maximum retention should be employed. The current paper describes a simple technique to record flabby tissues in their undisplaced state using readily available clinical materials like polyvinylsiloxanes in varying consistencies. The advantage of choosing monophase impression material is that, due to the inherent nature of the material, different consistencies can be achieved by varying the pressure applied on the material during mixing. Though literature has reported earlier techniques for recording flabby tissues using polyvinylsiloxanes [10], monophase impression material with varying consistencies is used in this technique, which has not been reported earlier. Use of impression compound as a stopper for an edentulous mandibular impression also has its advantages as it avoids placing conventional stoppers in areas which are flabby. The presence of impression compound also helps in orienting the tray thereby ensuring proper seating during border moulding and secondary impression procedures. Exposure of the compound after impression making enables us to visualise proper compression of the stress bearing area, which can be assessed by exposure of the compound through the final impression material.

## 4. Conclusion

The two impression techniques described in this paper requires the same amount of appointments as required for the fabrication of the normal complete denture. The materials are also readily available in the market and the method is easy to master and also does not require elaborate time, equipment, and auxiliary personnel thereby making it easy to be incorporated in our day to day dental practice.
